# Rethinking Postdischarge Intervention Evaluation

**DOI:** 10.2196/98435

**Published:** 2026-05-07

**Authors:** Brigitte Fong Yeong Woo

**Affiliations:** 1 Nursing Administration, Ng Teng Fong General Hospital, 1 Jurong East Street 21, Singapore, 609606, Singapore, 65 91803325

**Keywords:** postdischarge care, telephone calls, health service evaluation, patient readmission, hospitalization, emergency department, continuity of care, digital health

## Abstract

This commentary argues that for low-intensity postdischarge interventions, emergency department use may be a more sensitive and appropriate indicator of transitional care quality than readmission. It also positions nurse-led telephone follow-up as interpretive, equity-sensitive transitional care work that helps patients make discharge plans actionable in the home context while highlighting the value of accessible, scalable digital modalities such as telephone outreach.

Du et al [1] reported that a nurse-led telephone call delivered approximately 48 hours after discharge was associated with lower emergency department (ED) use at 7 and 30 days but without statistically significant reductions in hospital readmission. Rather than interpreting this as a partial success, I would argue that the pattern of findings raises a more important conceptual point: for low-intensity postdischarge interventions, ED use may be a more appropriate and sensitive indicator of transitional care quality than readmission alone.

This argument matters because the hospital readmission metric continues to dominate how postdischarge interventions are judged, despite longstanding concerns about its conceptual and methodological limitations. Readmission is influenced not only by discharge quality but also by disease severity, frailty, multimorbidity, caregiver capacity, community service availability, and broader social disadvantage. A recent commentary in *JAMA Network Open* [2] has further argued that an exclusive focus on 30-day readmissions is increasingly inadequate because it omits other clinically meaningful revisits, particularly ED presentations and observation stays, which constitute a substantial proportion of postdischarge acute care use. Earlier policy analyses have similarly questioned the reliability of readmission rates as stand-alone indicators of hospital quality [3]. Against that background, Du et al’s [1] findings may be less surprising than they first appear. A single follow-up call may reasonably reduce uncertainty-driven ED attendance without being sufficiently intensive to alter the more complex causal pathways that produce rehospitalization.

This interpretation is also consistent with the broader transitional care literature. A recent systematic review of interventions supporting transition from acute care to home in older adults found that benefits are most often observed with multicomponent interventions, especially those combining early follow-up, family involvement, and multidisciplinary input, and even then, effects tend to be modest and short-term [4]. Put differently, readmission is not an outcome that can be expected to shift easily in response to a single contact point, however well designed. When readmission becomes the dominant evaluative standard, there is a risk that lower-intensity but operationally scalable interventions are undervalued, even when they meaningfully improve continuity, reassurance, symptom management, or service navigation.

A second contribution of Du et al’s [1] study is that it draws attention to the mechanism by which seemingly modest interventions may work. The authors found that 40% of completed calls identified at least one gap in discharge understanding or follow-up care. This finding should be interpreted in light of the wider evidence showing that patient understanding at discharge is often overestimated by clinicians and by health systems. Studies in emergency and hospital settings have repeatedly shown deficits in patients’ comprehension of diagnosis, medication instructions, follow-up plans, and return precautions [5]. Recent work on discharge quality has similarly suggested that written instructions, while important, do not eliminate inequities in preparedness or self-management capacity after hospitalization [6]. The implication is that postdischarge follow-up should not be viewed simply as reinforcement of information already given. It functions as a second-stage clinical assessment of whether the discharge plan is intelligible and actionable in the patient’s actual home context.

From a nursing perspective, this is highly significant. Transitional care is often framed administratively, but the telephone call in this study is better understood as interpretive clinical work. The nurse is not merely checking whether instructions were received; the nurse is assessing whether they can be operationalized, whether medication regimens are manageable, whether symptoms are being interpreted appropriately, and whether follow-up arrangements are viable. This aligns with longstanding evidence that transitional care is most effective when it addresses coordination, continuity, and patient capability rather than information transfer alone [7]. In that respect, Du et al [1] contribute to a more realistic account of what postdischarge nursing support accomplishes: it manages uncertainty at the boundary between institutional care and patient self-management.

The paper is also relevant to current debates in digital health. Du et al [1] explicitly position telephone outreach as a digital health intervention. That framing is important because the digital health literature increasingly recognizes that technologically complex interventions can widen inequities when access, literacy, language, and infrastructure are unevenly distributed. A recent scoping review concluded that digital health technologies can reinforce inequalities and called for implementation strategies that explicitly address digital inclusion [8]. Empirical studies of telemedicine use have likewise shown that older age, limited English proficiency, public insurance, and other markers of disadvantage are associated with lower uptake of video visits relative to telephone care [9]. The significance of the intervention studied by Du et al [1], therefore, is not merely that it was effective but that it was delivered through a modality with comparatively low access barriers. In an environment where digital innovation is often equated with increasing technical sophistication, this study usefully supports a different principle: the value of a digital intervention should be judged partly by its reach and success in implementation, not only by its novelty.

The study’s quasi-randomized, operational design also deserves attention. As it was embedded in routine service delivery, the findings speak to effectiveness under real service conditions rather than efficacy under idealized trial circumstances [1]. That does not remove concerns about allocation, exposure, or residual confounding, but it increases the policy relevance of the findings. The more important question is whether a service can be standardized, integrated into existing workflows, and delivered at scale without excessive burden. On that question, this study is informative.

As such, the central insight from Du et al [1] is not simply that a nurse-led telephone call reduces ED use. It is that evaluation of transitional care may need recalibration. If the intervention target is postdischarge self-management, then ED use may be the more responsive outcome because it captures a form of potentially preventable acute care use that precedes or substitutes for readmission. Future work should, therefore, move beyond a binary success-or-failure assessment based on readmission and instead evaluate transitional care using a broader set of outcomes, including ED revisits, patient preparedness, unresolved care gaps, and the distribution of benefit across patient groups. That approach would be more aligned with both the complexity of discharge and the realities of contemporary health system use.

## Abbreviations

ED: emergency department
